# Role of IL-27 in COVID-19: A Thin Line between Protection and Disease Promotion

**DOI:** 10.3390/ijms25147953

**Published:** 2024-07-21

**Authors:** Zoia R. Korobova, Natalia A. Arsentieva, Angela Santoni, Areg A. Totolian

**Affiliations:** 1 Laboratory of Molecular Immunology, Saint Petersburg Pasteur Institute, 197101 Saint Petersburg, Russia; zoia-korobova@yandex.ru (Z.R.K.);; 2 Department of Immunology, Pavlov First State Medical University of Saint Petersburg, 197022 Saint Petersburg, Russia; 3 Department of Molecular Medicine, Pasteur Institute–Cenci Bolognetti Foundation, Sapienza University of Rome, 00162 Rome, Italy

**Keywords:** cytokines, IL-27, COVID-19, cellular immunity, inflammation

## Abstract

Cytokine storm is usually described as one of the main reasons behind COVID-associated mortality. Cytokines are essential protein molecules engaged in immune responses; they play a critical role in protection against infections. However, they also contribute to inflammatory reactions and tissue damage, becoming a double-edged sword in the context of COVID-19. Recent studies have suggested various cytokines and chemokines that play a crucial role in the immune response to SARS-CoV-2 infection. One such cytokine is interleukin 27 (IL-27), which has been found to be elevated in the blood plasma of patients with COVID-19. Within this study, we will explore the role of IL-27 in immune responses and analyze both the existing literature and our own prior research findings on this cytokine in the context of COVID-19. It affects a wide variety of immune cells. Regardless of the pathological process it is involved in, IL-27 is critical for upholding the necessary balance between tissue damage and cytotoxicity against infectious agents and/or tumors. In COVID-19, it is involved in multiple processes, including antiviral cytotoxicity via CD8+ cells, IgG subclass switching, and even the activation of Tregs.

## 1. Introduction

The COVID-19 pandemic has caused a global health crisis, with millions of confirmed cases and deaths worldwide [1]. COVID-19 is caused by the SARS-CoV-2 virus, an RNA virus of the *Betacoronavirus* genus, which enters the body through the respiratory system and attaches to ACE2 (angiotensin-converting enzyme II). This receptor is widely present on cells in different parts of the body: the respiratory system, gastrointestinal tract, blood vessels, myocardial and pericardial tissues, on the thyroid gland, epididymis, pancreas, liver, and even placenta [2]. Upon entry, the virus initiates immune reactions, which can cause inflammatory responses to become hyperergic and cascade-like. This hyperinflammation has the potential to cause a cytokine storm and widespread inflammation throughout the body, resulting in tissue damage to various organs such as the respiratory tract, heart, kidneys, and nervous system [3]. Cytokine storm is usually described as one of the main reasons behind COVID-associated mortality [4].

The virus primarily affects the respiratory system, and the symptoms of infection vary in their severity, from the common cold to pneumonia with severe acute respiratory syndrome [5]. However, the pathophysiology behind COVID-19 is rather complex, as it involves multiple organ systems, including the immune system.

Cytokines are essential small protein molecules of molecular mass < 25 kDa. They are engaged in immune responses and play a critical role in the regulation of multiple processes, including protection against infections. However, they also contribute to inflammatory reactions and tissue damage, becoming a double-edged sword in the context of COVID-19. Recent studies have suggested various cytokines and chemokines play a crucial role in the immune response to SARS-CoV-2 infection. One such cytokine is interleukin 27 (IL-27), which has been found to be elevated in the blood plasma of patients with COVID-19.

Within this study, we will explore the role of IL-27 in immune responses and analyze both the existing literature and our own prior research findings on this cytokine in the context of COVID-19.

## 2. Interleukin 27 Conformation and Its Biological Function in Immunity

IL-27 consists of two subunits, spiral-shaped IL-27 protein 28 (IL-27p28) and soluble Epstein–Barr virus-induced gene 3 (EBI3), which combine to form a heterodimer. It belongs to a larger family of dimer cytokines, including interleukins 12, 23 and 35 (IL-12, IL-23, and IL-35) [6]. IL-27p28 is the α-subunit with a four-helix bundle structure, consisting of 243 amino acids (aa), and it is encoded on human chromosome 16. EBI3 is the β-subunit, a protein made up of 229 aa, which is found on human chromosome 19. The EBI3 subunit combined with IL12p35 creates another interleukin, IL-35, which is involved in the function of T regulatory cells (Tregs) and B regulatory cells (B regs) [7]. IL-27p28 (also known as IL-30) was initially discovered as an orphan homologue of the interleukin 6/12 (IL-6/12) family in 2002 through a computational analysis of expressed sequence tags [8]. In contrast, EBI3 was first identified in 1996 as an erythropoietin receptor-like protein induced by Epstein–Barr virus infection in B cells [9]. Antigen-presenting cells typically express both subunits of IL-27, and the secretion of human p28 is reliant on the secretion of human EBI3. However, in mice, p28 can be secreted separately without relying on other factors [10]. The receptor for IL-27 (IL-27R) is also a heterodimer molecule. It is composed of two subunits: IL-27 receptor alpha (IL-27Rα) and glycoprotein 130 (gp130). IL-27Rα is unique to IL-27R in the IL-6/IL-12 family, and gp130 is also a subunit of the IL-6 receptor and interleukin 35 (IL-35) receptor [11]. IL-27p28 links IL-27Rα and gp130 to start signaling, with EBI3 stabilizing the complex by binding to specific regions of the receptors [12]. The IL-27 receptor is present on various cell types, such as dendritic cells (DCs), monocytes, macrophages, neutrophils, mast cells, eosinophils, T, B, and natural killer (NK) cells.

The interleukin 30 (IL-30)/EBI3 combination, known as IL-27, has both regulatory and anti-inflammatory functions. At first, it was believed to be similar to IL-12, which promotes T helper type 1 (Th1) immunity by inducing T-box transcription factor (T-bet) and IL12R-beta-2 expression through signal transducer and activator of transcription 1 (STAT1) activation during Th1 differentiation [13]. However, it was later found that IL-27 also has a strong inhibitory effect during T helper type 17 (Th17) differentiation [14].

When interacting with its receptor, IL-27 uses a STAT1-dependent mechanism to stop activated T cells from developing retinoic acid receptor-related orphan receptor (ROR-gamma-t) and interleukin 17 (IL-17) expression [15]. IL-27 also functions to suppress T helper type 2 (Th2) immunity through the inhibition of Th2 cell differentiation and subsequent cytokine production, as well as by directly suppressing type 2 innate lymphoid cells (ILC2s) [16]. IL-27 is involved in suppression of T regs activity. It suppresses Transforming Growth Factor-Beta (TFG-β)-induced Protein Foxp3+ regulatory T cell differentiation. Moreover, even one subunit of this heterodimer cytokine (p28) can affect Tregs’ generation and promote CD4+ activation [17]. IL-27 exerts anti-inflammatory functions by directly acting on conventional CD4 T cells to induce the differentiation of interleukin 10 (IL-10)-producing cells, which are a subset of regulatory T cells that play a critical role in controlling inflammatory responses and maintaining immune homeostasis [18].

**Figure 1:**
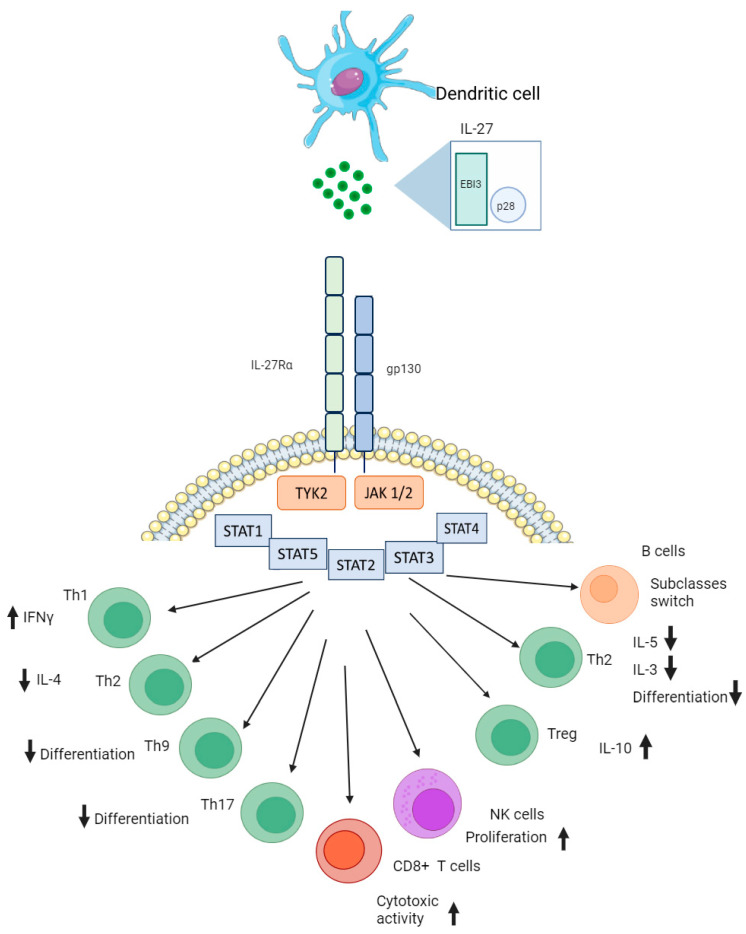
Simplified role of interleukin 27 in immune responses. Produced by dendritic cells, this cytokine serves multiple purposes, as it affects the IL-27 receptor on CD4+, CD8+, B, and natural killer cells. Abbreviations: EBI3—Epstein–Barr virus-induced gene 3, Gp130—glycoprotein 130, IL-27Rα—interleukin 27 receptor alpha, JAK1/2—Janus kinase 1 or 2, NK cells—natural killer cells, P28—protein 28, STAT—signal transducer and activator of transcription, Th—T helper cell, TYK2—non-receptor tyrosine-protein kinase. Adapted from ref. [22,29]. For visualization, we used OpenSource web platforms Photopea (https://photopea.com (accessed on 23 June 2024)) and Bioicons (https://bioicons.com (accessed on 23 June 2024)).

IL-27 can also function in collaboration with other cytokines, including members of its own family, IL-6, and others, IL-10, interleukin-11 (IL-11), and interferons (FNs) [19,20,21]. When collaborating with IL-6, it enhanced the promotion of local T regulatory cells and controlled Th1-mediated immunity [19]. Additionally, when the IL27p28 subunit pairs up with cytokine-like factor-1 (CLF-1), it forms a functional heterodimeric molecule that affects T cells and NK cells [22]. The immunomodulatory effects of IL-27 on a number of adaptive and innate immune cells are presented in Figure 1. On the spectrum between pro- and anti-inflammatory effects, it tends to lean towards suppression of inflammatory processes [23].

IL-27 affects hemopoiesis by activating stem cells. When combined with interleukins 15 and 18 (IL-15 and IL-18), IL-27 boosts the growth of NKs and increases their production of IFN-γ, granzyme B, and perforin. IL-27 signaling enhances the growth of CD4+ T cells, CD8+ T cells, and B cells. It also boosts the activity of T-bet and encourages the development of Th1 cells by upregulating IL-12 receptor β (IL-12Rβ2) expression. However, later, IL-27 signaling inhibits the production of IL-2, resulting in the suppression of Th1 cell differentiation [19].

For IL-27, both pro-inflammatory and anti-inflammatory properties are mediated by the phosphorylation of STAT1 and STAT3 molecules and their subsequent activation. However, its pro-inflammatory effects rely on the induction of T-bet and IL-12Rβ2 expression. IL-27 has been found to have a dual function in preventing tissue damage caused by excessive inflammation. Its impact on NK cells and their ability to regulate tumor growth has been observed in certain murine models, but not in all of them, suggesting that the effects of IL-27 on mouse NK cells may vary depending on the type of tumor [23]. In other words, IL-27 has a varying spectrum of functions, and it may be difficult to predict the overall effects of this cytokine, as its functional activity relies on several factors, such as signaling pathways and target cells.

In oncology, IL-27 is often discussed as a potential agent capable of stimulating anti-tumor immunity [24]. Various preclinical murine models have provided evidence that IL-27 shows strong antitumor effects against different types of tumors. These effects are achieved through several mechanisms and do not seem to cause any negative effects. These mechanisms involve recruitment of CD8+ T cells, natural killer cells, and macrophages, as well as antibody-dependent cell-mediated cytotoxicity and inhibition of cyclooxygenase-2 and prostaglandin E2 expression. These factors have direct effects on tumor cells, including inhibition of cell proliferation, tumor cell apoptosis, and suppression of epithelial–mesenchymal transition [20].

However, anti-tumor immunity is rarely achieved through the activity of only IL-27. In a review by Kourko et al., IL-27 effects are investigated in combination with other cytokines (IL-30, IL-35). Interactions of these so-called ‘sister’ cytokines have the ability to hinder the development of tumors by interacting with cancer cells either directly or indirectly, by activating various types of immune cells. For instance, IL-27 and other cytokines increase NK cell-mediated cytotoxic activity, promote the cytotoxic activity of CD8+ effector T cells, and stimulate the generation of CD8+ memory T cells. At the same time, IL-27 provides certain pro-tumor effects. It is produced by tumor cells and can inhibit chemotherapeutic effects [25]. When tumor cells undergo apoptosis, they are processed via endocytosis by antigen-presenting cells, which stimulate regulatory T cell activation [26].

Overall, in the context of cancer, IL-27 is mostly discussed as a pro-inflammatory cytokine that stimulates anti-tumor effects via activation of tumor-specific cytotoxicity. Unlike the stimulation of tumor immunity, IL-27 is often suggested as a therapeutic option in autoimmune diseases because it has anti-inflammatory effects.

Another important area of research on IL-27 is autoimmunity and autoimmune diseases. In vivo studies of autoimmune encephalomyelitis showed that the introduction of IL-27 affected Th17 cells and suppressed autoinflammation [27]. In a review by Meka et al., IL-27 is described as one of the most important cytokines in autoimmune diseases. In rheumatoid arthritis, IL-27 is often found in the synovial tissue of affected joints. IL-27 targets antigen-presenting cells to reduce the release of inflammatory cytokines like IL-1β, IL-6, and IL-23. It can directly block the development of Th17 cells and indirectly suppress their activity by boosting Th1 response. Moreover, IL-27 promotes the generation of regulatory T cells and IL-10, shifting the balance towards immune regulation over inflammation. It also hampers the production of matrix metalloproteinases (MMPs) and VEGF in various cell types, curbing angiogenesis. Additionally, IL-27 limits the production of receptor activator of nuclear factor kappa-B ligand (RANKL), a key factor in osteoclast formation, leading to decreased arthritic inflammation. Injections of IL-27 cause disease improvement when injected in a murine model of rheumatoid arthritis. Overall, a similar picture is seen for other autoimmune pathologies, i.e., systemic lupus erythematosus, colitis, psoriasis, and diabetes mellitus [28].

As mentioned above, IL-27 can play an ambiguous role in terms of inflammatory reactions. It is known to augment antigen-specific generation of cytotoxic lymphocytes [29], specifically via activation of T-bet and Eomesodermin/Tbr2 (EOMES). In addition, IL-27 plays a key role in the generation of Th1 immune responses. IL-27 is produced by the antigen-presenting cells upon activation and promotes the rapid proliferation of CD4+ T cells that have previously encountered the antigen [10]. It also works together with IL-12 to induce IFN-γ production by CD4+ T cells. However, IL-27 does not have the same effect on memory CD4+ T cells. Lucas et al. showed that IL-27 is insufficient to stimulate full-scale IFN production by the innate cells, but it plays a contributing role in Th1 responses [30]. At the same time, IL-27 suppresses Th2 cell differentiation by blocking the expression of GATA-3, a transcription factor deeply involved in Th2 responses [31]. In a study conducted by Schneider R. et al., it was demonstrated that IL-27 presence empowered CD8+ T cell proliferation and their development into T cytotoxic (Tc1) cells [32]; IL-27 promotes the expansion of self-renewing CD8+ T cells [33].

Overall, IL-27 interacts with cells via the IL-27 receptor and activates or inhibits their activity through STAT1/3 signaling molecules.

## 3. Interleukin 27 in Infectious Pathology

Due to the abovementioned diversity of IL-27 effects, its role in infectious diseases is often a matter of debate. It launches pro-inflammatory and protective effects by stimulating Th1-mediated immune responses. At the same time, it elicits regulatory effects by causing the expression of co-inhibitory receptors on T cells, including programmed death ligand 1 (PD-L1), lymphocyte-activation gene 3 (LAG-3), T-cell immunoglobulin and mucin domain 3 (TIM-3), cytotoxic T-lymphocyte-associated protein 4 (CTLA-4), and T cell immunoreceptor with Ig and ITIM domains (TIGIT) [28]. During infections, IL-27 levels are usually high. This tendency is associated with the expression of IL-10, which could create a mechanism for controlling inflammation while still allowing the infectious agent to thrive [34].

In bacterial infections, especially those that are often followed by sepsis, IL-27 is usually elevated in the blood plasma [35]. In several studies, it has been suggested that IL-27 can serve as a marker of prognosis in sepsis [36,37]. After IL-27 signal blockade by a newly synthesized protein, the survival rates in murine models of sepsis increased [38]. However, several studies have demonstrated that IL-27 is a poor choice for a prognostic biomarker in sepsis-associated conditions [39,40]. The effects of IL-27 in sepsis also relied on IL-27 gene polymorphisms: the *IL27 -964A > G* polymorphism increased the expression of IL-27 and exacerbated inflammatory responses in sepsis, leading to the advancement of sepsis and a negative outcome [41].

The same tendency was noted in *Mycobacterium tuberculosis* infection (TB): IL-27 showed elevated concentrations in the plasma of patients with tuberculosis when compared to healthy donors [42]. Elevated levels of this cytokine were also detected in pleural fluid and sputum [43], which can be associated with local inflammatory processes within the lung tissue. In one of the studies concerning TB, IL-27 was named ‘a wolf in sheep’s clothing’ due to the fact that in its absence, better control of mycobacterium growth was noted, but at the same time, its absence resulted in chronic hyperinflammation in later stages [44]. The accumulation of the intracellular EBI3 subunit of IL-27 in virulent *M. tuberculosis*-treated macrophages led to apoptosis inhibition [45]. In another study on *M. tuberculosis*, it was shown that IL-27 inhibits the anti-mycobacterial activity of macrophages [46].

A respiratory *Chlamydia muridarum* infection study performed by Zha et al. showed that IL-27 stimulates Th1-mediated inflammatory responses and affects neutrophil activation, mediating protective immunity against this infection [47].

In *Staphilococcus aureus* infection models, IL-27 receptor α knock-out mice were less susceptible to the infection than wild-type mice [48]. In this study, mice were previously infected with influenza H1N1 and exposed to *S. aureus* only after that.

In influenza, IL-27 also plays an ambiguous role. In murine experiments conducted by Liu et al., IL-27 was found to limit immunopathology and neutrophil accumulation, and it dampened Th1 or Th17 responses via IL-10-dependent and -independent pathways. In the absence of IL-27 signals, a more severe disease course was observed [49]. In the murine model by Jiang et al., IL-27 and IL-2 supported the expression of two transcription factors, IFN regulatory factor 4 (IRF4) and B-lymphocyte-induced maturation protein 1 (Blimp1). These transcription factors are essential for the generation of IL-10 by active CD8+ T cells [50]. In HIV infection, IL-27 has varying effects on gene expression in CD4 T cells and macrophage-derived chemokines [51]. It triggers the expression of antiviral genes in macrophages, as does IFNα. Therefore, IL-27 may hinder HIV replication in macrophage-derived cells through mechanisms resembling those of IFNα. In the study by Zheng et al., levels of plasma IL-27 were positively correlated with levels of gp130 CD4+ T cells in patients who underwent antiretroviral therapy [52]. In chronic viral infections, IL-27 is a key player in viral clearance. Intrinsic gp130-IL27 signaling promotes the survival of virus-specific CD4+ T cells. Deleting the gp130 receptor in T cells significantly reduced the survival of virus-specific CD4+ T cells, T follicular helper responses, and IL-21 production during the late stages of chronic lymphocytic choriomeningitis virus (LCMV) infection. This had a negative impact on CD8+ T cells, antibodies, and viral control. IL-27, as well as other gp130 cytokines, played a crucial role in promoting antiviral CD4+ T cell accumulation and inducing IL-21 [53]. The results indicate that IL-27 plays a crucial role in regulating immunopathology, and that administering IL-27 at appropriate times could be an effective treatment for severe inflammation caused by infections.

At the same time, in parasitic infections (i.e., *Leishmania donovani*), Th1 responses mediated by IL-27 signaling are directed towards the limitation of inflammation [54]. IL-27 suppressed glycolysis in inflammatory CD4+ T cells, leading to slower expansion of leishmaniasis. Without IL-27 signaling, the cells proliferated more quickly to combat parasite growth, but this also resulted in tissue damage in the spleen.

## 4. Interleukin 27 in COVID-19

As shown in other pathologies, IL-27 demonstrates a diversity of functions. We believe IL-27 to be a potential key player in the development of COVID-19 and protection against it. There are several studies concerning the involvement of IL-27 in COVID-associated immunity. IL-27 is a protein that is produced by immune cells when they are activated by pathogens or TLR ligands. It can stimulate the release of inflammatory cytokines from various types of cells, including mast cells, monocytes, and keratinocytes [55].

Initially, our investigation was aimed at analyzing the role of this cytokine in COVID-19 patients. Upon comparing acute-phase COVID-19 patients with healthy donors, we observed a statistically significant increase in IL-27 plasma levels in acute COVID-19 patients. Conversely, we also noted an opposite trend in convalescent patients, where concentrations of IL-27 were lower than those in healthy donors [56]. This tendency was seen independently of disease severity in the first-wave COVID patients in early 2020. The study included 56 patients aged 27–85 in the acute stage of the disease, with a gender distribution of 50% women and 50% men, all of whom were previously unvaccinated and free from co-infection or chronic diseases in the stage of re-emergence.

In a study performed by Zamani et al., IL-27 was proposed as a potential predictor for the severity and outcome of COVID-19 [57], along with IL-32 and neutrophil-to-lymphocyte ratio (NLR). The study included 50 COVID-19 patients, evenly split between non-severe and severe cases, who exhibited symptoms for at least 3–5 days from March to December 2021, with exclusion criteria for certain health conditions and immunosuppressive treatment.

In a study by Klingler et al., IL-27 was identified as a factor indicating the necessity for hospitalization in patients who have a higher risk due to demographic factors such as being male, of black or Hispanic ethnicity, and having a median age over 63 years [58]. For this research, 52 samples from COVID-19 survivors were divided into two groups: 10 hospitalized and 42 ambulatory. The median age of hospitalized patients was 68, compared to 62 for ambulatory patients. Approximately 40% of patients in both groups had comorbidities, but information on vaccination status was not provided.

Another study used machine learning to predict the outcome of COVID-19 and identified a few cytokines, including IL-27, IL-9, IL-12p40, and monocyte-chemotactic protein 3 (MCP-3), as potential indicators for mild cases of the disease [59]. Eighty-seven individuals with COVID-19 were hospitalized from March to May 2020 and were assessed based on symptoms and medical history. Severe cases required oxygen therapy, whereas non-severe cases did not. Patients with chronic conditions or recent platelet-disrupting medication use were not included in the study, and none had been vaccinated due to the early stage of the pandemic.

The 2023 publication authored by Valdés-López and Urcuqui-Inchima revealed that the identification of mRNA encoding IL-27 subunits in peripheral blood mononuclear cells (PBMCs) and monocytes from individuals with COVID-19 was linked to the severity of the disease [60]. This analysis of mRNA datasets included data from 13 healthy individuals and patients with moderate (*n* = 11), severe (*n* = 10), or critical (*n* = 11) COVID-19. Monocytes from nine healthy individuals and patients with moderate (*n* = 9) or severe (*n* = 11) COVID-19 were also studied. Participants ranged in age from 18 to 80 years old, with those having unstable chronic disorders or bacterial co-infections excluded from the study. Additionally, mRNA expression levels of IL-27 signaling components in COVID-derived PBMCs showed positive correlations between the expression level of mRNA encoding the IL-27p28 subunit and IL-27 signaling components in COVID-derived PBMCs, including Gp130, JAK2, STAT1, STAT3, and suppressor of cytokine signaling 3 (SOCS3).

In addition to our previous studies on the role of cytokines in COVID-19, we conducted another investigation to explore the association between IL-27 blood plasma concentrations and disease outcome [61]. All patients in the study were initially infected with the original Wuhan strain, and no vaccines were available at the time. The patients included were 58% male and 42% female, with ages ranging from 31 to 83 (average age 62.1 ± 12.5). They were divided into two groups: survivors (46% male, 54% female), with ages from 31 to 77 (average age 55.8 ± 13.6); and non-survivors (69% male, 31% female), with ages from 45 to 83. The median age for non-survivors was 65 ± 16.0, whereas for survivors, it was 53 ± 14.4. Upon hospital admission, 72.4% of individuals (21 patients) had a moderate course of the disease, and 27.6% (eight patients) had severe infection. The assessment of the disease course was based on Russian Ministry of Healthcare guidelines for COVID-19 treatment. Our research showed that there was a significant increase in concentrations among both COVID-19 survivors and non-survivors when compared to healthy individuals. However, there was no statistical significance found when comparing the survivors and non-survivors. And though it is possible that IL-27 may have predictive value, our analysis showed no sufficient sensitivity or specificity to support this claim. We used receiver operating characteristic analysis to assess the predictive value of IL-27 and found that it was not a reliable marker for prognosis.

In studies concerning other respiratory pathologies, IL-27 has shown a positive correlation with the severity of pulmonary inflammation [62]. The study included 58 patients suffering from acute lung injury/acute respiratory distress syndrome, with an average age of 46 years, consisting of 36 males and 22 females. Average APACHE II (Acute Physiology and Chronic Health Evaluation II) score comprised 17.8. In 239 patients diagnosed with community-associated pneumonia (CAP), serum levels of IL-27 were elevated in comparison with healthy donors [63].

However, as we delved further into the investigation of IL-27 and its involvement in COVID-19, we also analyzed the correlation between IL-27 and the SARS-CoV-2 genetic variant. In one of our studies, we performed multiplex cytokine profiling in plasma samples from patients infected with different SARS-CoV-2 variants (i.e., 2019-nCoV strain or Wuhan strain, B.1.1.7 or Alpha variant, B.1.617.2 or Delta variant, and B.1.1.529 or Omicron variant) [64]. The study included a total of 289 participants who were experiencing their first COVID-19 infection, had not received any prior vaccination, and fell within the age range of 32 to 90 years. Our results suggest that IL-27 plasma levels are not a reliable indicator of the severity of COVID-19 infection, as they do consistently vary with the viral variant. However, the four cytokines (IL-6, IL-10, IL-18, and IL-27) that were consistently elevated in COVID-19 patients may provide valuable insights into the equilibrium behind disease progression and host infection. Further research is needed to confirm these findings and determine their clinical significance.

When analyzing CD8+ T cell subpopulations in acute COVID-19 patients, convalescents, and healthy donors, we noted a negative correlation with the CCR6+CD8+ T cell frequencies within effector memory T cells and terminally differentiated effector memory T cells re-expressing CD45RA in patients with acute COVID-19 [65]. This tendency was not evident in COVID-19 convalescents or healthy controls. Moreover, serum IL-27 levels also showed a negative correlation with the absolute numbers of central memory and terminally differentiated effector memory cells (Tc17 cells) from patients with acute COVID-19 but not from convalescent or healthy donors. This study examined 71 individuals with acute COVID-19, 51 convalescent subjects with serum SARS-CoV-2 N-protein-specific IgG antibodies, and 46 healthy volunteers without detectable antibodies to any SARS-CoV-2 proteins. The research was conducted between May and November 2020. The average age of the participants in this group was 60 years old, with a gender ratio of approximately 35.2% males and 64.8% females.

In the study by Schneider R. et al., IL-27 presence was affecting CD8+ T cell proliferation and inducing their development into Tc1 cells [32].

All findings concerning IL-27 in COVID-19 are presented in Table 1.

**Figure 2:**
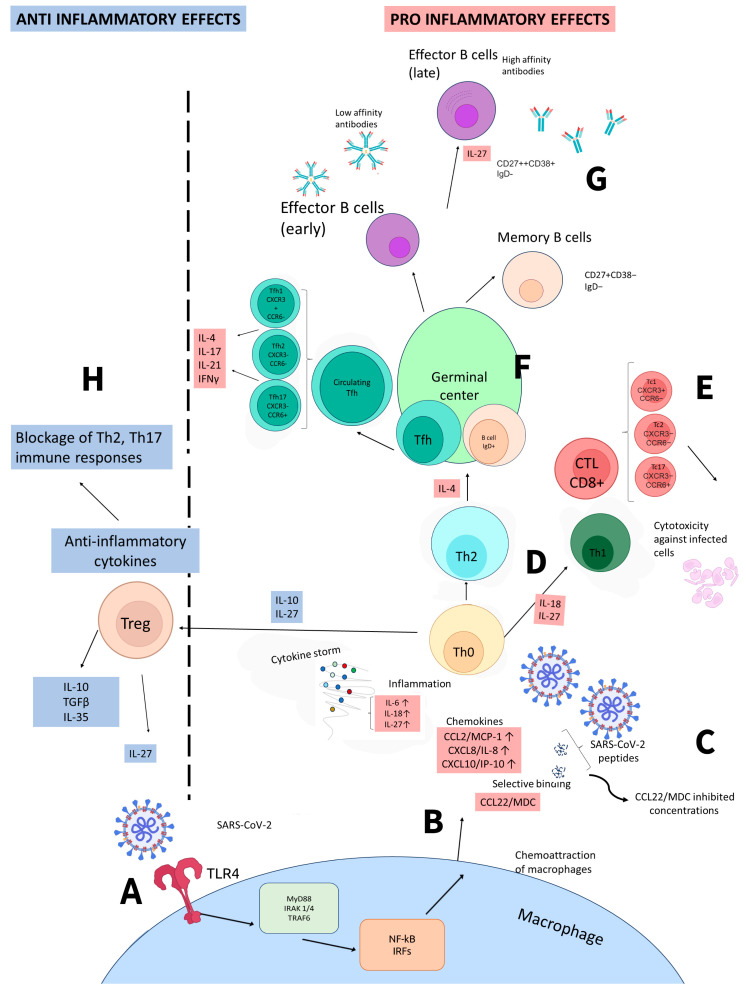
Simplified representation of immune responses to SARS-CoV-2 and the outcomes associated with them. (**A**) SARS-CoV-2 interacts with innate immune cells (e.g., macrophages) via the TLR4 receptor. Subsequent signaling leads to the production of cytokines and chemokines. (**B**) Immune cells produce pro-inflammatory cytokines, IFNs, and chemokines that attract other immune cells. (**C**) IFN γ also stimulates the production of IFN-dependent cytokines and chemokines, such as CXCL10/IP-10. In its presence, in addition to the influence of viral SARS-CoV-2 peptides, the production of MDC/CCL22 by macrophages decreases, which leads to a depletion in concentrations of this chemokine, as noted in our studies earlier. (**D**) Upon cytokine production, Th0 differentiation branches out depending on the cytokines involved, including Th1-mediated immune responses with further production of IFN γ and involvement of macrophages. (**E**) Involvement of cytotoxic lymphocytes (CTLs) in immune responses is also mediated by IL-27. It leads to cytotoxicity toward infected cells. (**F**) In the germinal center, via activation of Th2 immune responses and IL-4 production, B cells differentiate into effector plasma cells or memory B cells. (**G**) Plasma cells produce antigen-specific antibodies, whereas IL-27 is involved in subclass switching. (**H**) anti-inflammatory effects of IL-27 are affecting the differentiation of T regulatory cells. IL-27 is also involved in switching Th2, Th17 immune responses towards anti-viral Th1 immunity (B-E). Abbreviations: CCL2—C-C chemokine motif ligand 2, CCR6—C-C chemokine receptor type 6, CXCL10—C-X-C chemokine motif ligand 10, CXCL8—C-X-C chemokine motif ligand 8, CXCR3—C-X-C chemokine receptor type 3, IFN—interferon, IL—interleukin, IRAK 1/4—interleukin-1 receptor-associated kinase or 4, IRFs—interferon regulatory factors, MDC—macrophage-derived chemokine, MyD88—myeloid differentiation primary response gene (88), NF-kB—nuclear factor NF-kappa-B, Tc—T cytotoxic lymphocyte, Tfh—T follicular helper cells, Th—T helper cell, TLR4—Toll-like receptor 4, TRAF6—TNF receptor-associated factor 6. For visualization, we used OpenSource web platforms Photopea (https://photopea.com) and Bioicons (https://bioicons.com).

IL-27, among other cytokines, is deeply involved in immune-mediated processes associated with COVID-19. As its receptor is widely present on a variety of cells, its function is strongly interconnected with other cytokines and chemokines. We present our view on how IL-27 is integrated into immune processes in COVID-19 in Figure 2.

## 5. Conclusions

Interleukin 27 is a cytokine endowed with diverse functional activities. It can play both pro- and anti-inflammatory roles, depending on the signaling pathway and the target cells it affects. Overall, regardless of the pathological process it is involved in, IL-27 is critical for upholding the necessary balance between tissue damage and cytotoxicity against infectious agents and/or tumors.

In COVID-19, its role is also ambiguous. As one of the few cytokines that shows statistically significant changes in concentrations in the acute phase of the infection, IL-27 is directly linked to the markers of cell-mediated immunity. And even though its full role in the immune responses behind COVID-19 is yet to be explored, it is now clear that IL-27 may be one of the most interesting cytokines involved in this infection.

## 6. Future Directions

We are open to discussing this topic further and welcome any insights or perspectives that may help us better understand the role of IL-27 and other cytokines in COVID-19. By gaining a better understanding of the immune response to SARS-CoV-2, we hope to identify new therapeutic targets and develop more effective treatments for this devastating disease.

## Figures and Tables

**Table 1:** Research overview on the role of IL-27 in COVID-19.

Year of Publication	Authors	Findings	Reference
2021	Arsentieva N.A. et al.	Increase in IL-27 levels in acute COVID-19 patients vs. healthy donors (*p* < 0.0001); decrease in IL-27 concentrations in convalescents when compared to healthy donors (*p* = 0.0015).	[56]
2022	Arsentieva N.A. et al.	IL-27 showed a statistically significant increase in concentrations in COVID-19 acute patients when compared to healthy donors (*p* < 0.001 for non-survivors and *p* < 0.05 for survivors).	[61]
2022	Korobova Z.R. et al.	IL-27 was one of the four biological markers showing statistically significant changes in concentrations in the blood plasma of patients infected with different variants of SARS-CoV-2 (*p* < 0.001).	[64]
2022	Kudryavtsev I.V. et al.	The correlation between Tc17 cells of central memory and TEMRA cells and serum IL-27 levels was negative in patients with acute COVID-19, a tendency not observed in convalescent or healthy donors.	[65]
2022	Zamani B. et al.	IL-27, along with IL-32 and neutrophil-to-lymphocyte ratio (NLR), was highlighted as one of the markers of severe COVID-19 and lethal outcomes.	[57]
2022	Klingler J. et al.	In patients with higher demographic risk factors (i.e., male, black/Hispanic descent, and median age over 63 years old), IL-27 was suggested as one of the factors to prove the need for hospitalization.	[58]
2023	Laatifi M. et al.	The COVID-19 prognosis was based on machine learning and included several cytokines, including IL-27, along with IL-9, IL-12p40, and MCP-3, to play the role of markers for non-severe COVID-19.	[60]
2023	Valdés-López and Urcuqui-Inchima	IL-27 triggers a strong pro-inflammatory and antiviral reaction that relies on STAT1 without requiring IFN in COVID-19-derived PBMCs and monocytes, which is linked to a severe clinical outcome of COVID-19. This effect is also seen in macrophages that have been stimulated by S protein.	[62]

## Data Availability

Data sharing is not applicable.
